# Assessing the impact of specialist home visiting upon maltreatment in England: a feasibility study of data linkage from a public health trial to routine health and social care data

**DOI:** 10.1186/s40814-018-0294-4

**Published:** 2018-06-28

**Authors:** Fiona Lugg-Widger, Rebecca Cannings-John, Lianna Angel, Gwenllian Moody, Jeremy Segrott, Joyce Kenkre, Michael Robling

**Affiliations:** 1 Centre for Trials Research, Cardiff University, Neuadd Meirionnydd, Heath Park, Cardiff, CF14 4YS UK; 2 DECIPHer Centre, Cardiff University, Cardiff, UK; 3 Faculty of Life Sciences and Education, University of South Wales, Pontypridd, UK

**Keywords:** Routine data, Feasibility study, Progression criteria, Teenage mothers, Home visiting, Child maltreatment

## Abstract

**Background:**

Follow-up for public health trials may benefit from greater use of routine data. Our trial of a home-visiting intervention for first-time teenage mothers assessed outcomes to the child’s second birthday. To examine its medium-term impact, particularly upon maltreatment outcomes, we designed a study using routine records.

**Methods:**

We aimed to establish the feasibility of our study design, which combines trial data with routine health, social care and education data using a dissent-based linkage model. Trial participant identifiers were linked to routine health, social care and education data if women did not dissent. Data were forwarded to a safe haven and further linked to de-identified trial outcome data. The feasibility study aimed first to establish the acceptability of data linkage through a discussion group of young mothers and by levels of dissent received by the research team. Second, we assessed levels of accurate linkage to both health (via NHS Digital) and education and social care (both via National Pupil Database, NPD). Third, we assessed the availability of data and levels of missingness for key outcomes received for a sample of target study years.

**Results:**

Of 1545 mother-child dyads contacted, eight women opted out. The engagement exercise with stakeholders found support for the principle of data linkage, including in the context of maltreatment. Some contributors preferred opt-in consent. Most (99.9%) health records were matched on either three or all four identifiers. Fifty participants were not matched to any health data. Primary outcome data from NPD are derived from any one of three fields, all of which were satisfactorily returned and provided an indication of cases for analysis. Missing data for secondary outcomes varied from 0% (Child looked after status) to 70% (Anatomical Area A&E diagnosis) however when combined with other variables the levels of missingness for outcome decrease.

**Conclusions:**

Through study set-up and in this pilot, we provide evidence that the main study is feasible, satisfies governance requirements and is likely to generate data of sufficient quality to address our main research questions. Observed levels of missingness or low event rates are likely to affect some secondary analysis (e.g. state transition modelling) although overall were satisfactory.

**Electronic supplementary material:**

The online version of this article (10.1186/s40814-018-0294-4) contains supplementary material, which is available to authorized users.

## Background

Achieving a successful start in life can be particularly challenging for children born to teenage mothers who themselves may struggle to achieve longer term socio-economic stability [1, 2]. We have previously reported on the Building Blocks (BB:0–2) trial of a nurse-led home-visiting intervention, the Family Nurse Partnership (FNP) being delivered to teenage first-time mothers living in 18 sites in England [3–6]. Over 1600 women participated in the trial, which randomly allocated women to either usually available supportive health and social care alone (usual care) or visits provided in addition to usual care from specially trained FNP nurses from the end of the first trimester until their first child was aged 2 years.

Large-scale evaluations of community-based home-visiting and similar public health interventions present a number of challenges. Adverse socio-economic circumstances facing families may create barriers to identifying and recruiting women in the first place and retaining engagement over an extended period of time can impact outcome assessment. For example, while our self-report follow-up rate at 24 months was 70%, in two contemporary trials of the same or similar intervention in the Netherlands and in Germany, the rates were 48% and 46% respectively [7, 8]. The BB:0–2 trial also made use of routinely provided healthcare data which was used solely or in combination with other data for both primary and secondary outcomes, enabling more data to be available for analysis compared with just self-report data.

In BB:0–2, families were followed up at 24 months post-partum but the programme was expected to impart beneficial effects on child health and development and maternal life course that would accrue many years after visiting ended. These benefits would be expected to extend into multiple sectors such as education and social care, and so beyond the original primarily healthcare setting of the intervention. Key outcomes would include domains that are sensitive in nature such as maltreatment, which may be subject to reporting bias and non-response bias if solely assessed by maternal self-report.

Therefore, we designed a study which used routine data to evaluate longer term programme impact [9]. This seeks to create a pseudonymised (i.e. replaces key characteristics in the dataset so that individuals cannot be directly identified) research database comprising the original trial dataset with a further 4 years of data from health, social care and education records. Unlike the original BB:0–2 trial, which involved prospectively recorded participant consent, this study would require a dissent process and no additional recourse to self-reported data. As the BB:0–2 trial made use of routinely collected data, we had some reassurance about the feasibility of using some of the expected data for longer term evaluation but not all data sources and not with the dissent model.

Key remaining questions about the viability of the research design were addressed through a two-stage pilot. The first stage summarised the integrity of programme delivery, potential effect and ability to access the routinely collected data. This was in response to funder review comments requiring these elements to be addressed early on in the study and treated as start-stop criteria for the continuation of the study. The second stage addressed a range of feasibility parameters related to participant identification, matching and record linkage and data quality, including missingness and numbers available for analysis. Criteria thresholds for progression were not set; rather the project team reported findings to their management group and independent steering committee for information. The rationale for this second stage was to ensure the final datasets could answer the research questions robustly and timely. Records supplied via the Department for Education’s National Pupil Database include those from several linked datasets including safeguarding data from local authority departments of children’s social care, a primary outcome for the study. The data providers for our study use different unique identifiers to match (e.g. NHS Digital primarily use NHS number, National Pupil Database use name, postcode, date of birth and gender). When we collected baseline trial data, we were intending to solely link to healthcare records. How well these identifiers from hard to reach young families could be used to allow us to develop a research database of sufficient coverage and data quality needed to be verified. Finally, our research plan involves unconsented access to identifiable records for families who had previously consented to trial participation. We considered that we needed to explore the views of similar members of the public (acting as stakeholders) about such activity and its acceptability (to note, not directly with participants). The aim of this paper is to describe our study to establish the feasibility of using trial data linked to records from multiple sources to study the longer term impact of a specialist home visiting programme to support teenage first-time mothers in England.

## Methods

The study design of this follow-on study using routinely collected data has been described previously in a published protocol paper [9]. For convenience, we briefly summarise the essential design here before describing the methods specific to the pilot phase evaluation.

### Overview of study design

Building Blocks:2–6 (BB:2–6) aims to extend the duration of follow-up for participants exiting the BB:0–2 trial of the Family Nurse Partnership intervention [3]. It will do so by identifying and linking to routine data from three principal sources, NHS Digital (health data), the National Pupil Database (NPD; education and select social care data) and the Office of National Statistics (ONS; mortality data). These data will be matched to participant identifiers held by the trial team at Cardiff University. The two primary data centres, NHS Digital and the NPD, use a different combination of identifiers for matching. NHS Digital use NHS numbers, date of birth, postcode and gender. The NPD uses a combination of forename, surname, date of birth and postcode. Both data centres use exact matching, with NPD fuzzy matching by forename if required. Unique pupil number—a unique matching variable used by NPD—was not collected during the trial however will be assigned following matching to enable linkage across NPD datasets.

Retrieved data minus personal identifiers will be provided direct to a trusted third party data safe haven which will use the project-specific identifiers to link these data to trial outcome data sent from the trial team. Project identifiers are removed and replaced with an encrypted anonymised linking field (ALF-E). Data are accessible to named researchers via a secured remote portal. Data are processed legally under section 251 approval provided via the Confidentiality Advisory Group, Health Research Authority [10]. Trial participants were offered the opportunity to dissent from the study following contacts made by post, email and text messages.

### Two-stage pilot

Evidence to support progression of the study (pilot stage one) was gathered via a number of sources. Some of these related to conduct of the original trial (specifically adequacy of intervention delivery and of short-term effect) and are briefly summarised in Additional file 1. The key feasibility elements addressed in the second pilot stage were (i) developing an adequate participant dissent model, (ii) establishing acceptable levels of record linkage and (iii) establishing adequate data quality. The governance model outlining required approvals has been described in the BB:2–6 protocol paper [9]. The full follow-up period for the study will include records to 31 March 2017, representing the end of the six-year follow-up. For the pilot stage 2 we requested data from centres to enable a sufficiently informative assessment of data linkage and quality. For NHS Digital (and ONS mortality data which is accessed via the same provider), this included data from study entry of the first mother (June 2009) to 31 March 2015. Local authority safeguarding data accessed via the modular NPD datasets were requested to 31 March 2014 and education data requested from NPD to other end-points in 2014 (Table 1).

**Table 1:** Data requested for the second pilot stage

Provided by	Dataset	Eligibility/coverage		Mother	Child	Requested for the pilot
Dept. of Health	Abortions	England and Wales All abortions performed in the NHS or an approved independent sector		✓	✗	✗
ONS	Mortality records	UK		✓	✓	Entry–31 March 2015
NHS Digital	Inpatient	Any NHS hospital in England		✓	✓	Entry–31 March 2015
	Outpatient					Entry–31 March 2015
	Accident and Emergency					Entry–31 March 2015
Dept. for Education	Child In Need	< 18 years registered with social services in England		✓	✓	Entry–31 March 2014
	Child Looked After					Entry–31 March 2014
	Early Years Foundation Stage Profile	Public schools in England	4 years	✗	✓	Assessment day July 2013 and July 2014
	Early Years Census		3–4 years	✗	✓	Census day Jan 2013 and Jan 2014
	Alternative Provision		2–19 years	✓	✓	Census day Jan 2013 and Jan 2014
	Pupil Referral Unit		2–19 years	✓	✓	Census day Jan 2014
	School census		2–19 years	✓	✓	Winter term 2012–Summer term 2014
	Key stage One		5–7 years	✗	✓	✗

#### (i) Developing and assessing adequacy of dissent model

There were two components to this assessment. First, we implemented the process that provided trial participants an opportunity to register their dissent. Dissent could be registered through a variety of channels (online, email, text message, phone, post). Early on in the study set-up, we worked with a group of care-experienced young people (i.e. have spent time in the care of the local authority) to develop our written letter to trial participants [11, 12]. Numbers of trial participants approached and numbers of dissenting responses received were recorded.

Through a public engagement/involvement process, we explored key factors which influenced the acceptability of the planned data linkage and the importance of anonymity to a contact group of young mothers, and how we could develop materials to support dissemination of study findings (and the research methods used) to interested lay parties. Two researchers (JS, JK) met with an on-going young mothers group (‘Our Place’) who had previously provided lay input to the Building Blocks trial [13] as external stakeholders (i.e. these were not trial participants). A plan for the meeting was jointly developed within the research team, including audio-recording this single session with the approximately 20 mothers, who were expected to attend the group’s own regular meeting place in South Wales. Verbal agreement from stakeholders was obtained prior to their participation in the session with the researchers. This was following previous communication between the research team and group coordinators including the provision of information to mothers in advance of the meeting. An initial discussion was held with the group as a whole and then the researchers worked with two smaller (self-selected) groups to gather their views of the use of linked datasets. The discussion was supported by the use of visual aids, which provided further information about the topic (e.g. A4 cards describing datasets being linked). These mothers were also not participants of research, instead external stakeholders. Although the output from the discussions are presented descriptively in line with topic headings, this public involvement was not undertaken as qualitative research and no formal qualitative methodology was applied.

#### (ii) Establishing acceptable record linkage

The number and proportion of participant identifiers matched to records by each data centre was assessed. For NHS Digital data, this also included an assessment of the match rate by each step in the matching algorithm (Table 2). For both data centres, matches would include both mothers and children. We also assessed descriptively the process for receipt, de-identification and linkage of datasets by the data safe haven.

**Table 2:** NHS Digital match algorithm

Step (match rate)^a^	NHS number	DoB	Sex	Postcode
1	Exact	Exact	Exact	Exact
2	Exact	Exact	Exact	–
3	Exact	Partial	Exact	Exact
4	Exact	Partial	Exact	–
5	Exact	–	–	Exact
6^b^	–	Exact	Exact	Exact
7^c^	–	Exact	Exact	Exact
8	Exact	–	–	–

^a^Matching at step 1 or 2 would provide greatest reassurance of valid match

^b^Where NHS number does not contradict the match and DOB is not 1 January and the postcode is not in the ‘ignore’ list

^c^Where NHS number does not contradict the match and DOB is not 1 January

#### (iii) Establishing adequacy of data quality

We assessed data availability and completeness for all variables supplied from both data centres required for primary and secondary analyses. Priority was placed on primary and key secondary outcomes. Numbers of available records, reasons for missingness and narrative assessment of potential impact were undertaken to indicate potential feasibility of the main study.

## Results

### (i) Adequacy of dissent model

#### Retaining eligible participants

**Fig. 1:**
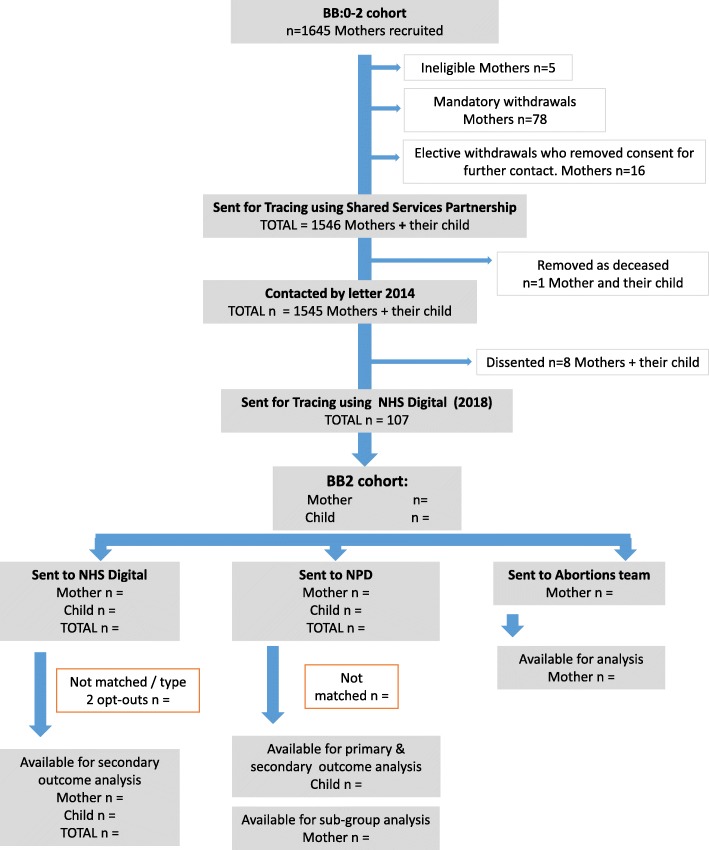
Participant flowchart: families recruited in BB:0–2 trial and followed up in BB:2–6 feasibility study

One mother and child dyad was removed due to a child death, leaving 1545 mother-child dyads to contact (Fig. 1). Of these, 93 had electively withdrawn during the original trial. In October 2014, letters were sent to all 1452 women who had not electively withdrawn and additionally, SMS text messages (*n* = 653, 45%) and emails (*n* = 386, 27%) to those women where contact details were available. Following additional approval to contact women who had electively withdrawn from the trial, we contacted all 93 women by letter in September 2016, and of these, we also sent text messages (*n* = 60, 65%) and emails (*n* = 16, 17%) where possible. Of the 1545 mothers contacted, eight (0.5%) dissented and were excluded from the research database. This was made up of seven and one from the 2014 and 2016 letters respectively. Additional approval was required from ethics and the confidentiality advisory group for the withdrawn population to ensure the letter sent to them reflected that they had previously withdrawn from the trial.

#### Stakeholder views on linkage

Audio-recording of the discussion with Our Place mothers proved impractical due to background noise, and contemporaneous notes were taken instead. Twenty mothers were in attendance at the meeting and their children so two groups with six and five mothers each spent time separate to the main group with the researchers to discuss data linkage and its use in more detail. Representing the data linkage process using A3 sheets (for organisations) and A4 sheets (for datasets) and how anonymity was preserved when data were accessed by the research team appeared to be informative for participants. The group expressed preferences for a greater use of visual methods (for example, using computers, pictures to represent organisations). The ease by which individuals could be identified through combining data across datasets arose as a question from the group.

Although focus group stakeholders were content with the data linkage procedure described and with reassurances about anonymity, there was nevertheless concern expressed about data security against hacking. The nature of data being held (e.g. more sensitive data on maltreatment) did not affect the perceived acceptability of the linkage approach. One participant asked about the possibility of individuals requesting their own data, which may suggest that there remained some lack of clarity about the non-reversibility of anonymisation. One important area where some disagreement within the group arose was the use the of dissent model. The group appeared to be mostly supportive of this approach given the original consent provided in the preceding trial, the efforts made by the researchers to contact women and the pseudonymisation of data involved. However, some participants preferred an opt-in approach as a general principle.

### (ii) Adequacy of data linkage

Match rates to NHS Digital and NPD datasets are shown in Tables 3 and 4. For NHS Digital, 2851 unique records were sent and 2804 (98.4%) participants were matched, of these, 2801 participants (99.9%) were matched at either step 1 or 2 (see Table 2 for definition) indicating a greater reassurance of matching to correct individual. There were 64 participants (31 mothers, 33 children) missing from the Inpatients dataset where they would have been expected (i.e. as there should be at least a birth record). However, 15 of these were present in other NHS Digital datasets, indicating a successful match but missing an inpatient record. Forty-nine participants did not appear in any dataset, which is likely due to matching failure or National opt-out (whereby NHS patients in England electively opt-out of their clinical data being used for purposes other than their own direct care).

**Table 3:** Match (any type of match) rates for NHS Digital and NPD

	Participants sent (*n*)	Participants matched (*n*)	Proportion matched (%)
NHS Digital			
Mother	1434	1407	98.1
Child	1419	1397	98.4
NPD			
Mother	1428	99	6.9
Child	1412	1272	90

**Table 4:** Data received from NHS Digital

Dataset name	*N* participants in dataset	*N* mothers in dataset	*N* children in dataset	*N* match steps 1 and 2 (% based on *N* participants in dataset)	*N* records in dataset (multiple records per participant)
A&E	2451	1205	1246	2446 (99.8%)	13,211
Outpatients	2338	1398	940	2336 (99.9%)	39,067
Inpatients	2789	1403^a^	1386^b^	2786 (99.9%)	11,882

^a^31 missing, 27 unmatched and 4 of these present in A&E dataset

^b^33 missing, 22 unmatched and 11 of these present in A&E and Outpatients datasets

For NPD data, mothers would appear only if aged under 19 years and a child in need or looked after, or in school (Table 5). The denominator for planned study primary outcome analysis would be the 90% of children adequately matched. The number of records matched per NPD dataset reflected age of children and duration of coverage of each requested dataset.

**Table 5:** Data received from National Pupil Database (NPD)

NPD dataset name	Years provided	Records in dataset (*n*)	Participants in dataset (*n*)	Mothers in dataset (*n*)	Children in dataset (*n*)
Pupil Level and School Census (PLASC)	2012/2013; 2013/2014	760	760	4	756^a^
Pupil Referral Unit (PRU) Census	2013/2014	2	2	2	0
Alternative Provision	2012/2013; 2013/2014	1	1	0	1
Early Years Census (EYC)	2012/2013; 2013/2014	581	565	0	565^a^
Child Looked After (CLA)	2008/2009–2013/2014	23	23	10	13
Child in Need	2008/2009–2013/2014	331^b^	169^b^	98^b^	71

^a^54% of 1412 children were identified in PLASC; 40% in EYC. Summer 2014 was the last school census dataset requested for the pilot thus not all children would have been expected to be in school (i.e. only by March 2014, would all children have turned 3 years of age)

^b^1 record received does not contain any data and therefore following further data cleaning may be removed

### (iii) Adequacy of data quality

Assessment included establishing that key outcomes could be adequately derived from supplied data. The primary study outcome is Child in Need (CIN) status to be derived from a combination of three NPD CIN dataset fields (Referral date, Referral but no further action, Reason for closure). For these and all fields retrieved, we undertook an impact assessment to clarify the field’s role in analysis, number of records retrieved, explanatory notes regarding missingness and impact on planned analysis. A field’s purpose would include acting as primary or secondary outcome (either in combination or with other fields), for cross-checking/validation of other data and for planned exploratory analysis. Impact was assessed as either No, Low, Medium, High or Not required, with explanations where justified.

A summary version of the final assessment table is shown to demonstrate these key elements and how that informed the feasibility assessment for each variable (Table 6). The primary outcome of Child in Need status being recorded by age 6 years is determined from three fields in the NPD Child In Need dataset which shows referral date, further action taken and closure date within the reporting year. A return in any one of these three fields would indicate a positive CIN status. As records would only appear in this dataset following a conditional event (i.e. a referral), it is not possible to assess absent valid cases but does indicate potential number of cases for inclusion in the main analyses. Other secondary outcomes are similarly formed of several fields both for NPD data (e.g. Child protection registration) and NHS Digital data (e.g. Injuries and ingestions) and presence can be inferred by positive entries in one or more of the contributing fields. Levels of missingness in current pilot and original trial data matching are shown where relevant. Some planned analyses were found to be potentially affected by level of missing data (e.g. state transition modelling) or small numbers (Child Looked after status), which would either reduce the scope of analysis or indicate a descriptive approach respectively. Many of the fields in the HES data that show high levels of missing data will be combined (e.g. diagnosis and treatment) and therefore where there is a value in one of these fields it would be assumed that this was an event within the A&E dataset. Missing data may also make some outcomes difficult to derive. In these cases, any assumptions made on the missing information will be stated and if possible varied (worse /best case scenario) and caveats will be made around results to aid interpretation.

**Table 6:** Outcomes and data fields assessed in pilot: records available and feasibility assessment

Outcomes	Data source: *native field name*	Missing (*n*)	Commentary of findings	Impact
*Primary*				
Child in Need (CIN) status as of 31 March each year	NPD > CIN: *Referral date*	1	No data appear across one record—record to be excluded (this will apply to all fields below) Note: 34 records with dates prior to time point ranges (1997–2007). This is expected.	None
	NPD > CIN: *Referral—no further action*	42	No data collected in 2008/2009 time point (accounts for 38 records). Some blanks appear in 2009/2010 time point; however, the referral date on these records is prior to 1st April of that data collection year. *Assumption that time point cycle is Apr–Mar.	Low: assumption missing data indicate further action was required and that the child was in need
	NPD > CIN: *Reason for closure*	142	No pattern—further investigation required	As above
*Secondary* ^a^				
CIN categorisation	NPD > CIN: *Category of Abuse*	329	Only data from 2008/2009 accessed in pilot. For main phase data from 2012/2003 will be accessed and also ‘NPD > CIN: Latest category of abuse’ will be included which may improve data quality.	Still to be determined
Child looked after (CLA) status (1)	NPD > CLA: *Category of need; Legal status; Placement; REC*	0	All records returned are complete.	None
Child Protection registration (plan) (1)	NPD > CIN: *Child Protection Plan (CPP) Indicator*	195	Expected—all missing from 2010/2011 time point onwards. Data not collected during these years.	Low: CPP flag can be determined from other fields
	NPD > CIN: *No. of previous Child Protection Plans*	320	No pattern to missingness. Only 11 records have a value recorded, 9 of these are zero.	As above
	NPD > CIN: *Child Protection Plan start date*	320	Expected—not all children will have had a CPP. Only 11 records have a date recorded, these correspond with data captured in the ‘no. of previous CPPs’ above.	As above
	NPD > CIN: *Child Protection Plan end date*	327	Expected—only 4 records have an end date recorded. Corresponds with those records where a start date is recorded. Data check done—end dates are after the start date.	As above
Exploratory Markov chain modelling^b^	NPD > CIN: *Date of initial child protection conference*	327	Expected—not all children would have had a child protection conference. However further checks required to confirm validity of data.	Medium: Low numbers may impact analysis
Injuries and ingestions	NHSD > A&E: *A&E diagnosis (diag n)*	5981	45% missing (1650/6336 missing in BB trial—26% missing)	Medium: All diag/treat/inv. fields to be used in combination to define inj/ing^c^
	NHSD > A&E: *A&E diagnosis*—*2 char (diag2 n D)*	3604	27% missing (1849/6336 missing in BB trial—29% missing)	As above
	NHSD > A&E: *A&E investigation (invest n)*	1728	13% missing (1396/6336 missing in BB trial—22% missing)	As above
	NHSD > A&E: *A&E investigation*—*2 char (invest n D)*	1712	13% missing (1395/6336 missing in BB trial—22% missing)	As above
	NHSD > A&E: *A&E treatment (treat n)*	2349	18% missing (1411/6336 missing in BB trial—22% missing)	As above
	NHSD>A&E: *A&E treatment – 2 Char (treat2 n D)*	2126	16% missing (1417/6336 missing in BB trial—22% missing)	As above
	NHSD > A&E: *A&E diagnosis*—*Anatomical Area (diaga n D)*	9281	70% missing (4725/6336 missing in BB trial—74% missing)	

(1) small numbers may be an issue—descriptive analysis will be used if necessary

^a^Additional fields were retrieved for secondary outcomes and assessed solely for presence (Special Educational Needs, Disability, Day care attendance, Early Years assessment, School attendance, Key stage one attainment)

^b^To explore probability of progression through each stage of child protection process

^c^Same fields also contribute to assessment of subsequent pregnancies (via pregnancy-related A&E attendances)

### Additional work

Data management protocols including de-identification for processing data from project team to data centres and collation at data safe haven were also tested. This included ensuring that the multiple datasets created by each of the two primary data centres could be re-combined while project identifiers known to the project team could be safely removed before data was made available to researchers. Standard data cleaning activities and data re-structuring were enacted but are not otherwise described here.

## Discussion

In this feasibility study, we tested a dissent process, which resulted in few trial participants dissenting, and then proceeded to match their identifiers to a high proportion of routine records. The latter include health data matched with a high level of precision using NHS Digital’s stepped algorithm process. Fields used in combination will form individual outcomes for the study limiting the impact of some apparent missingness. Some record matching had higher levels of missingness than observed for the same participants in the trial. Nevertheless, the primary outcome analysis appears feasible, as do analyses of many planned secondary outcomes. Low rates of some outcomes may indicate descriptive analysis only, and one of the planned analyses of state transition through phases of the child protection process will be limited by the reduced set of fields ultimately available.

We have established feasibility over two stages. The first required evidence that the evaluation of the nurse-visiting programme had been delivered with sufficient fidelity in the trial phase. A longer term evaluation also needed to be justified by some indication that the programme was at least not harmful. Progression criteria were developed in discussion with the funder, and the data gathered in the trial’s process and outcome evaluation respectively met these criteria. In a second stage, perhaps the most critical set of criteria addressed a range of feasibility parameters many of which could only be determined after the study set-up and through the pilot study presented here. The independent study steering committee has been essential in confirming the scope of, and then progression against these criteria. An inability to meet the criteria at stage one would have probably and correctly led to study closure. It is also possible that serious challenges in the second stage would result in the same decision. However, re-configuring our approach within the same study design was probably the more likely outcome. In practice, this is what has happened. Our analysis plan has been adjusted based on what we understand is likely to be available for analysis. The work undertaken to establish the governance infrastructure, mapping and managing the required data linkages and preparing datasets for main analysis (e.g. scripts for data cleaning and re-structuring) provides reassurance for the main study phase. There is greater emphasis being placed on clarity of objectives for feasibility and pilot trials, with detailed criteria and thresholds for progression [14]. Our study adds to that literature with its particular focus on unconsented data linkage from multiple data centres following up from a closed trial sample. Furthermore, we have developed a model for representing data flow relevant to this study type (Fig. 1) that will provide the basis for our main result presentation.

Our study comprises a number of strengths. We have developed and tested the mostly complete model of data linkage required for the main study, with the key data providers and ultimate data safe haven included. This allows us to draw more informed conclusions about how the final model of data linkage will work in concert to produce a viable research database. We have also used actual data from our intended study sample as the basis for the assessment as opposed to simply modelling using dummy data. This therefore provides a more direct test of matching quality and also likely available data, for example, levels of missingness. We have not presented the data in a way for study results to be interpreted; however, Table 4 does describe the number of records found for the cohort. The numbers presented here are consistent with the BB:0–2 trial with regard to the number of A&E attendance and admissions [3]. In addition, by testing the approach through actual data provider governance systems, we have been able to ensure that the final dataset can be assembled in a manner that remains acceptable to key stakeholders. Our work with the lay advisory panel has been supportive in this regard too.

Nevertheless, some questions remain of either direct or general importance. As some study data are events that may not occur for families (e.g. child protection referrals) the assumption is that the absence of a record from the dataset is a confirmation that there was no event, which may not be the case. Nevertheless, the presence of other related data for each family can be used to confidently infer an event in some cases and overall rates of data linkage remain high across both health and education data providers. The sample of years used for this pilot provides reassurance of what may be available when all years are subsequently requested. However, assessing education data reliant upon children reaching a certain schooling age means that we are currently less able to determine the quality and availability of data required in the main analysis.

Our impact assessment placed a greater focus on those variables contributing to primary and key secondary outcomes (e.g. Child in need). We needed to ensure that our main study question could be answered even if some other objectives were at risk. Data may be lost due to a variety of reasons, which also vary by contributing data centre—out of date identifiers (e.g. post code), opt-outs which are general (National opt-out) or specific to the study (dissents) and matching errors. Data may also be lost even before data are provided to the data centre (i.e. invalid returns to NHS Digital and NPD). The cumulative impact can only be fully assessed when the full dataset has been retrieved for the main study but our pilot sample provides a good estimate of what is possible.

An underpinning element of our study design is the extraction of routine data to be held pseudonymously in a third-party data safe haven and without direct consent. We were unable to obtain ethical approval in our original trial for long-term follow-up using routine data as the parameters for such data collection were not then fixed and the validity of baseline consent for much longer term consent was also questioned. We collected numerous contact details for all participants (including of key others, such as family members) at trial baseline, which were then periodically refreshed during the trial (during data collection and using a tracing service). While we cannot determine how complete actual notifications about the current study to all trial participants was in practice, this approach has helped to ensure that the process for capturing dissent is as meaningful and valid a process as possible.

We have explored how our general approach to accessing and using sensitive routine data is understood and judged by members of the public. There is considerable policy interest in routine data in research and some effort to align research practice with public opinion [15–17]. We explored how processes for linking and using data were understood and accepted by lay representatives. While we recognise that only a small number of mothers were involved, they still represented the population who are the subject of the intervention under evaluation. Importantly, we have identified topics for further exploration with the group, particularly dissemination. We will use this as the basis for developing materials to maximise public engagement with likely stakeholders and consumers of study results, including trial participants. We consider that it is incumbent upon researchers to consider the optimum role for public engagement in data linkage studies and proactively support this.

## Conclusions

Overall, we conclude that the main study objectives are achievable albeit that some secondary outcome analyses may be restricted by data that become available in the main data request phase. The value of public investment in similar trials can be increased through greater use of routine data, but questions of feasibility will still need to be answered. We have deployed a two-stage approach for decision-making on progression. The first stage may be characterised by decision options: progress, stop or substantially adjust, which in this scenario were mostly negotiated directly with the funder. A second stage may be characterised by decision options: progress, or adjust where possible with the steering committee (on behalf of the funder) and the project team negotiating progress. At this second stage, defining exact progression criteria may be less critical than simply understanding how available data have impacted upon study results and their interpretation.

## Additional file

Additional file 1: Evidence supporting progression derived from trial (BB:0–2) and feasibility study (BB: 2–6) phases. Detailed progression criteria as set out by the study funder at the start of the project to ensure research objectives could be met. (DOCX 35 kb)

## Abbreviations

ALF-E: Anonymised Linking Field (encrypted)
BB:0–2: Building Blocks trial
BB:2–6: Building Blocks: 2–6 study
CAG: Confidentiality Advisory Group
CIN: Child in Need
CLA: Child Looked After
EYC: Early Years Census
FNP: Family Nurse Partnership
HRA: Health Research Authority
NIHR PHR: National Institute for Health Research Public Health Research
NPD: National Pupil Database
ONS: Office for National Statistics
PLASC: Pupil Level and School Census
PRU: Pupil Referral Unit
