# Supplementary material for: Assessing the impact of specialist home visiting upon maltreatment in England: a feasibility study of data linkage from a public health trial to routine health and social care data
Source: Pilot Feasibility Stud. 2018 Jun 28;4:98. doi: 10.1186/s40814-018-0294-4 (PMC6022436; doi:10.1186/s40814-018-0294-4)
Supplement: Supplementary file 1 — Evidence supporting progression derived from trial (BB:0–2) and feasibility study (BB: 2–6) phases. Detailed progression criteria as set out by the study funder at the start of the project to ensure research objectives could be met. (DOCX 35 kb) [file 40814_2018_294_MOESM1_ESM.docx]

**Evidence supporting progression derived from trial (BB:0-2) and feasibility study (BB: 2-6) phases**

|  | ***Component(s)*** | ***Criteria*** | ***Notes*** | ***When evidenced*** |
| --- | --- | --- | --- | --- |
| ***1) Evidence of intervention delivery^1^*** | | |  |  |
| *FNP core model elements (CMEs)* | - Visiting regime, Staffing requirements, Client eligibility, Site bids and supporting organisation structure & processes | Aggregate evidence that CMEs meet specified licensing requirements. | Establishes intervention as adequately delivered and that outcome evaluation is justified | BB:0-2 process evaluation |
| *FNP fidelity stretch targets* | - Recruitment, Attrition, Dosage, Content | Aggregate evidence that specified stretch targets are being met. | Establishes intervention as adequately delivered and that outcome evaluation is justified | BB:0-2 process evaluation |
| ***2) Evidence of short-term effect^2^*** | | |  |  |
|  | *Injuries and ingestions* | Evidence of non-inferiority at age 2 years | Establishes intervention as not likely to be detrimental | BB:0-2 outcome evaluation |
| ***3) Evidence of feasibility*** | | |  |  |
| *Access approval* | HRA CAG approval | To have been obtained | Enables dissent model | BB:2-6 set-up |
|  | Social Care: ADCS^3^ approval | Informal support for submission | Not required due to modified data model via NPD | BB:2-6 set-up |
| *Dissent process* | Acceptability of dissent model | (i) Level of dissent expressed (quantitative)  (ii) Views from lay stakeholders on key principles |  | (i) BB:2-6 feasibility study  (ii) BB:2-6 feasibility study |
| *Data collection* | NHS: Retrieval of NHS Digital data | (i) Successful receipt in BB:0-2  (ii) Successful receipt in BB:2-6 |  | (i) BB:0-2  (ii) BB:2-6 feasibility study |
|  | NHS: Retrieval of GP data | Successful retrieval of participant data from manual GP extraction within BB:0-2  Confirmation of GPES^4^ terms of reference | Not required due to modified data model via NPD (although some secondary data missed) | BB:2-6 set-up |
| *Record linkage* | NHS data (all source) | (i) 90%+ linkage on existing for BB:0-2 study participant data sets  (ii) Levels of linkage for primary, key secondary outcomes in dissent model |  | (i) BB:0-2  (ii) BB:2-6 feasibility study |
|  |  | Overall feasibility of linking data |  | BB:2-6 feasibility study |
| *Data quality* | NHS data: Child in Need registration | (i) Assessment of missingness, validity  (ii) Assessment of missingness, data quality (primary and key secondary outcomes) | NHS data superseded by move to NPD as primary data source | (i) BB:2-6 set-up  (ii) BB:2-6 feasibility study |

*1 Established by process evaluation in BB:0-2 trial. Some details were only confirmable subsequent to funding award for BB:2-6 study.*

*2 Established by outcome evaluation in BB:0-2 trial. Only confirmable subsequent to funding award for BB:2-6 study*

*3 Association of Directors of Children’s Services*

*4 General Practice Extraction Service (NHS Digital)*
